# Supplementary material for: Effects of selenium nanoparticle on the growth performance and nutritional quality in Nile Tilapia, Oreochromis niloticus
Source: PLoS One. 2022 Jun 2;17(6):e0268348. doi: 10.1371/journal.pone.0268348 (PMC9162325; doi:10.1371/journal.pone.0268348)
Supplement: Attachment — Submitted filename: Tilapia manuscript.docx [file pone.0268348.s001.docx]

# Nano-Selenium supplementation improves the growth performance in Nile tilapia (*Oreochromis niloticus*) through the enhancement of w-3 fatty acids and nutrition quality index

Fasil Dawit Moges^1^, Hamida Hamdi**^2^, Amal Al-Barty^2^, Abeer Abu Zaid^3^, Manisha Sundaray^1^, S.K.S. Parashar^4^, Abebe Getahun Gubale^5^, Biswadeep Das^*1^

^1^ School of Biotechnology, Kalinga Institute of Industrial Technology, KIIT Deemed to Be University, Bhubaneswar, Odisha, India.

^2^Department of Biology, College of Science, Taif University, P.O. Box 11099, Taif 21944,

Saudi Arabia.

^3^Department of Biology, Alkhormah University College, Taif University, Taif 21974, Saudi Arabia.

^4^ School of Applied Science, Kalinga Institute of Industrial Technology, KIIT Deemed to Be University, Bhubaneswar, Odisha, India.

^5^ Department of Zoological Sciences, Addis Ababa University, Addis Ababa, Ethiopia.

*****Correspondence :

Biswadeep Das (Dr.)

[biswadeep.das@kiitbiotech.ac.in](mailto:biswadeep.das@kiitbiotech.ac.in)

**Co-Corresponding author

Shimaa76sl@tu.edu.sa

# Abstract

Selenium is an important micronutrient that has antioxidant, growth potential, and reproduction enhancement abilities in various organisms. At present, the aquaculture industry is under pressure to meet the dietary requirements of a third of the global population, thereby exploring approaches for enhancing the production of dietary fish. This study was performed to assess the growth performance of Nile tilapia (Oreochromis niloticus) fingerlings upon nano-selenium (Se-Nps) supplementation. Nanoselenium was synthesized using high-energy ball milling (HEBM) using a 10-hour dry milling technique at 10:1 ball-to-powder ratio (BPR), size characterized by XRD and TEM, followed by mixing with basal feed in desired concentrations (0.5 mg/kg, 1 mg/kg, and 2 mg/kg) and administration to Nile tilapia fingerlings for 30 days, followed by the evaluation of growth performance parameters, fatty acid profile analysis using GC-MS, and nutritional quality index (NQI): [Thrombogenicity Index (IT), Atherogenicity Index (IA), n-3/n-6, n-6/n-3)]. Nile tilapia supplemented with 1 mg/kg Se-Nps showed improved growth performance (RGR: 1576.04 %, SGR: 4.70 %, and FCR: 1.07 %), demonstrated by higher survivability (> 95%), isometric growth (coefficient of allometry, b = 2.81), and higher weight gain. The average length-weight relationship assessed as the condition factor (C) was highest in the 1 mg/kg N-Se group compared to others (p < 0.05). GC-MS analysis revealed that Nile tilapia supplemented with 1 mg/kg Se-Nps showed better flesh quality, higher amount of n-3 fatty acids, eicosapentaenoic acid, and docosahexaenoic acid, high PUFA/SAFA ratios (1.35) and n-3/n-6 (0.33) ratios, with low atherogenicity index (0.36) and thrombogenic index (0.44), and relatively low n-6/n-3 fatty acid ratio (3.00) compared to other groups. Overall, Se-Np supplementation at 1 mg/kg enhanced the growth performance and flesh quality in Nile tilapia thus could be a potential growth-promoting micronutrient for aquaculture production.

**Keywords:** Nanotechnology; Aquaculture; *Oreochromis niloticus*; Dietary Nano-Se; Growth Performance; Enriched Meat.

# 1. Introduction

The contribution of fish farming to global food requirements is huge owing to the increasing demand for fish as a primary nutritional source in several countries. The world aquaculture fish production reached 82.1 million tons in 2018, approximately 46% of the world’s fish production [1]. Nile tilapia (*Oreochromis niloticus*) is one of the commercially important fish, forming the basis of commercial fisheries in many parts of the world. Tilapia is a freshwater fish, which is one of the most widely reared aquatic fish across the world. The importance of Nile tilapia could be attributed to its versatile applications, including fast growth, ease of breeding in captivity, high productivity, high fecundity, major food source that caters to most human populations, competitive market price, stress and environment tolerant, and many others [2]. Tilapia culture has emerged as one of the most convenient and highly profitable fish farming methods in over 120 countries [3]. In 2017, tilapia was ranked as the fourth most popular culture fish and included in the top ten aquaculture-producing fish species groups across the world [4]. This trend coupled with the need to supply the world with more quality food is guiding researchers to explore various growth improvement techniques for tilapia culture [5].

Given, the importance of tilapia as a dietary and commercial fish across the world, information on the basic biology and aquaculture productivity of this species are crucial. The quantitative parameters of fish, such as condition factor, length-weight relationship, fecundity, and mortality are important aspects for the assessment of growth performance in natural environments [5, 6], and which have been applied to assess the effect of environmental alterations on the well-being of fish [6]. Growth in animals, including fish, is regarded as an improvement in either body size (weight, length, and girth) or health. The length-weight relationship provides information on the condition and growth performance of fish. Fish grow isometrically, when the length increases in equal proportion to body weight, whereas allometric growth is assumed when the increase in any of the parameters: length or weight is un-proportional to each other. Therefore, it is possible to estimate the weight or length of fish from either of each parameter that could be assessed using a formula, which takes into account the growth pattern: isometric or allometric [6, 7]. Condition factor is an index reflecting interactions between the biotic and abiotic factors with the physiological condition of fish. It has been demonstrated that heavier fish reflects a healthier physiological state [7]. Condition factor has been used as an indicator of health in fishing biology studies such as growth and feeding intensity. It can be used to determine the feeding activity of a species to determine whether it is making good use of its feeding source and obtaining sufficient energy through the diet [7].

Another feature for the assessment of fish quality is the type and variety of lipids and fatty acid composition in fish [8]. Fatty acids (FA) are one of the components of lipids or fats that are present in structures called triglycerides. FAs are either saturated (SFA) or unsaturated (UFA). In an SFA, the alkyl chain does not contain any double bonds, whereas UFAs contain one or more double bonds. Considerable variation occurs between the fat and lipid content of fish within and between species [8-11]. In general, fish is a healthy, easily digestible food, which is rich in UFAs and poly UFAs (PUFAs). The consumption of fish and fish lipids can provide PUFAs, in particular ω-3 PUFAs. PUFAs are known to diminish the level of blood cholesterol; therefore, they can prevent cardiovascular diseases [12]. Long-chain ω-3 PUFAs have to be obtained from the diet because they cannot be synthesized by humans [13]. Hence, obtaining essential fatty acids, such as docosahexaenoic acid (DHA) and eicosapentaenoic acid (EPA), and arachidonic acid (AA) from exogenous sources is very important for good health.

Dietary supplementation of micronutrients in the form of nanoparticles as feed supplements has been reported to enhance the productivity and meat quality of fish, e.g., nFe, nSe, nAg, and nZnO [14]. Nanosupplementation of micronutrients offers several advantages, such as greater bioavailability, easy absorption, enhanced utilization, and promoting cellular functions. Selenium is a trace element and has an important role in the development and immune status of organisms [15, 16]. Several researchers have conducted selenium supplementation for enhancing the productivity and growth performance in *Carassius auratus gibelio* [17], *Ctenopharyngodon idellus* [18], improved productivity and growth in stress-resilient fish [19] improved immune status in *Cyprinus carpio* [20] and juvenile *Lates* *Calcarifer* Block [21]. However, studies on the assessment of N-Se supplementation on the growth performance in tilapia are lacking. The present study assesses the growth performance, length-weight relationship, condition factor (FCF), fatty acid analysis and nutrition quality index, and dietary levels of Se-NP on the fingerlings of *O. niloticus* to improve the fish production and yield level and quality.

# 2. Materials and Methods

## 2.1. Synthesis and Characterization of Se-NPs

Selenium pellets of about 5 mm, > 99.99% trace metal basis were purchased from Sigma Aldrich, India. The synthesis of the dietary Se was performed in the Nano Lab., School of Applied Science, KIIT University, India using the High Energy Ball Milling (HEBM) through dry milling process according to our previous method [22]. Characterization of the synthesized Se-Nps was performed using XRD and TEM analysis, in addition to assessing the stability of the particle size distribution of the Se-NPs using a zeta sizer.

## 2.2. Tilapia rearing set up and breeding

All the methods were approved and carried out under relevant guidelines and regulations of the Institutional Animal Ethical Committee (IAEC) of Addis Ababa University (AAU), Ethiopia. Eight glass aquariums with 70*40*35 cm dimensions were set at the Green House-Based Research Recirculating Aquarium system facility (RAS), AAU based on the FAO RAS guideline [23]. Four filtering mechanisms were employed: mechanical (physical), biological, carbon filter, and UV light-based filtration were designed and set into three 250 L plastic barrels. A 2000 L plastic barrel was used for de-chlorinated water supply for the recirculating system and also as water backup. The dechlorinated and filtered water was pumped into the recirculating aquarium system using an electric water pump (0.5 HP). The utilized water flows back as overflow beyond the outlet level into the filtering mechanism starting the physical filtration using cotton pads. Unused feed and any physically visible wastes were siphoned using water pumps after an hour of every feeding. 2/3 of the overall recirculating water system was drained and the aquariums are cleaned every alternating day. Water quality parameters: temperature, pH, dissolved oxygen, and conductivity were maintained at optimum conditions. Parent stocks of Nile tilapia (*O. niloticus)*, were collected from the wild (Lake Chamo, Ethiopia). Healthy parents were selected and put into the brooding tank in the hatchery facility within the green-house. Brood-stock conditioning and stocking for breeding were done for brooder-parents at a ratio of 1:5 (Female to Male) for 12 days. At the end of the breeding days, each mother was checked for fertilized eggs. Eggs were collected manually from the mouth of brooding mothers and transferred into a bowl with dechlorinated water by carefully pressing the mouthparts of the mothers. The collected eggs were transferred to the hatchery set-up. The eggs were let in the recirculating hatchery system for a week.

## 2.3. Stocking and rearing experimental fishes

At the end of the week, the hatched fries with exhausted yolk sac were transferred to the nursery pond/tank. Feeding was provided with a fine size of basal feed only after their yolk sac was completely drained. The young fingerlings of the same cohort with an average body weight of 1.00 gm were randomly selected and stocked into the research RAS set-up at a stocking density of 50 fish per aquarium. The fries were acclimatized in the recirculating system for two weeks. Unhealthy fish and dead individuals were removed and the stocking density was reduced up to 40 healthy fish per aquarium. After completing the acclimatization period, the fish were raised for a total of eight weeks.

## 2.4. Feed formulation and nano-Se dietary supplementation

The basal feed was formulated with a calculated proximate composition of 35.06%, 32.54%, 12.00%, 18.00%, 2.00%, and 0.40% that corresponded to fish meal, soya bean, corn, wheat, CMC (carboxymethyl cellulose) sodium salt, and multivitamin, respectively. Dietary Se-NP was mixed with the basal feed using a fine powder mixer (IKA-EUROSTAR, power control visc) to make maximum homogenous treatments. The prepared control feeds and mixed experimental feed were transformed to very thick dough using distilled water. The dough was then extruded separately for each inclusion into pellets, followed by packing and storage at a cold store. The experimental supplement inclusions were: 0.5, 1.0, and 2.0 mg Se-Nps/Kg basal feed. Feeding of the experimental fish was performed using the experimental feed twice per day. After every hour of feeding, the remaining feed and waste pellets were siphoned to maintain a healthy and suitable culture medium.

## 2.5. Length-Weight relationship analysis

The length-weight relationship was calculated using the equation: W = a*L^b^

Where W is the weight gain in gm for every length L (TL) in cm. “a” and “b” are growth parameters. The wellbeing or the condition of the fish was calculated as FCF (Fulton’s Condition Factor):

$k=\frac{Weight (W)}{Length (L3)}$,

Where k is the condition factor (FCF), W is average weight (Av.) in gm, and L is length (Av. TL) in cm. To express the physical growth of tilapia, biometry indices were used. These biometry parameters were carried out at the beginning of the experiment every week, and at the end of the experiment. Bodyweight gain (BWG), specific growth rate (SGR), and relative growth rate (RGR) were calculated as:

*BWG = BWF- BWI--------equation 1,*

Where, BWG is weight gain (gm), BWF is the final weight in gm, and BWI is the initial body weight in gm.

$SGR=\frac{\left( ln BWF-\ln BWI \right)}{T}*100$*-------------equation 2,*

Where: SGR is specific growth rate and T is total cultured time.

$RGR \left( \% \right)=100*\frac{BWF-BWI}{IBW}$*-------------equation 3,*

Feed Conversion ratio, which is more related to growth performance, is calculated as

$FCR =\frac{Total Feed Provided (gm)}{Total Weight Gain (gm)}$*-------------equation 4.*

Where the total feed provided is the amount of feed used to raise live fish till the end of the experimental period, and the total weight gain is the difference in the biomass between the initial and the final weight.

The initial total weight for each treatment (WT) and length (TL) for the subsamples was measured on day one, followed by every 7th day of the week. At the end of the experiment (the 8th-week), growth parameter measurements and length-weight data were taken for each individual of all treatments. The total length of each fish was measured from the tip of the snout (mouth closed) to the end of the caudal fin using a measuring board. Blood from freshly cut gills and caudal fins, whole liver, and tissue samples were collected for Se level analysis. Moreover, tissues (muscle) collected before and after the experiments were used for FA profile analysis. Fish feed proximate analysis was also done separately. Dead fish were removed; if any, from each aquarium and weighed to calculate feed conversion ratio (FCR).

## 2.6. Gas Chromatography-Mass Spectrometry (GC-MS)

Gas Chromatography/Mass Spectrometry, or GC/MS analysis, is an analytical method that combines the features of gas chromatography and mass spectrometry to identify different substances; in this case FAs. GC/MS is used for the quantitative determination of fatty acids in foods and other sample matrices by assessing the conversion of fatty acids into methyl esters (FAME).

Briefly, FA profiling was done by isolating the lipid fraction through Soxhlet extraction using n-hexane for six hours. The lipid portion was then allowed to undergo saponification and derived to FAME (Fatty-acid Methyl Ester) by HCl/Methanol. Individual FAs profiling was done by Gas Chromatography (GC- USA Agilent 7890-B Technology) equipped with a split injector with a flow rate of 100 mL/min. The percentages of the individual FAs were determined using peak areas. Electron Ionization (EI) system was used to separate and quantify each FAME component. The FAMEs were separated using a highly polar HP-88 (USA, Agilent) column (30 m* 0.25 µmm* 0.20 µmm) in the Mass Spectroscopy detector (MSD- Agilent 5977-A). The carrier gas was helium (He) at a linear velocity of 1 ml/min. The oven temperature was 40 °C for 2 min and increased by 15 °C/min to 130 °C, at a rate 4^o^ C/min then to 150 °C, at a rate of 3 °C/min then to 160 ^o^ C held for 17 min, at a rate of 5 ^o^ C/min, to 205° C and finally at a rate of 15 ^o^ C/min to 230° C. Data analysis was performed by determining the percentage area of the FAs and the absolute concentration of specific FAs using external standards and NIST-17 Library (National Institute of Standards and Technology Standard Reference Data Program, Gaithersburg, MD 20899) using Agilent MassHunter (GC/MS, Acquisition: B.07.03.2129 18-Agilent Technologies, Inc.) software.

## 2.7. Nutritional quality indexes of lipids

The nutritional quality of the lipid portion of the samples was determined through the fatty acid compositions. This evaluation was performed using four nutritional quality indicators (indexes) based on fatty acid compositions. The atherogenicity and thrombogenicity indices were calculated according to [24], to assess the biological effects of different fatty acids:

**Atherogenicity Index (IA)**

$$IA=\frac{[(C 12:0+(4x C14:0) + 16:0) ]}{[ \sum MUFA+ \sum n6 + \sum n3]}$$

IA represents the relationship of the sum of the major saturated fatty acids to that of the major classes of unsaturated, the former being considered pro-atherogenic or pro-thrombotic, and the latter anti-atherogenic or anti-thrombotic, thereby preventing the appearance of micro-and macro- coronary diseases [24].

IA index relates to the content of SFAs with the content of MUFA and PUFAs w-3 and w-6. Generally, the consumption of foods/fish meat with a lower IA can reduce the level of total cholesterol and LDL-C (Low- Density Lipoprotein Cholesterol- ”Bad Cholesterol”) in human blood.

**Thrombogenicity Index (IT)**

$$IT=\frac{C 14:0 + C16 :0 + C18:0}{(0.5 x \sum MUFA)+(0.5 x \sum n6)+(3 x \sum n3)+\frac{\sum n3}{\sum n6}}$$

IT shows the tendency to form clots in the blood vessels. It relies on the data about the effect of several fatty acids on the plasma thrombogenic potential, indicating the tendency to form clots in the blood vessels. It represents the relationship of pro-thrombogenic FAs (C12:0, C14:0, and C16:0) and anti-thrombogenic FAs (MUFAs and the PUFA n-3 and PUFA n-6 families. Hence, the consumption of foods with low IT is beneficial for CVH Cardiovascular Health (CVH) [24].

# 3. Data Analysis

Data were analyzed using MS-Excel and the Statistical and Data Analysis Software Package: Minitab 17\18 (State College, PA: Minitab, Inc). Collected and obtained data were expressed as means ± SD for experiments independently. Shapiro Wilk test was performed on the data set to assess the normality of the distribution. A one-way analysis of variance (ANOVA) was performed to compare the difference in the respective mean values among the groups for all the assays followed by multiple comparison tests. Regression analysis was applied for the LWA (Length-weight Analysis). A P-value of < 0.05 was considered statistically significant.

# 4. Results

## 4.1. Characterization of synthesized Se Nanoparticles

Physico-chemical analysis revealed that the Se-Nps showed an average size distribution of 30-50 nm, and 40 ± 12 nm in aqueous solution (Fig. 1).

Figure 1. Physicochemical characterization showing the representative images of TEM **(a),** XRD **(b)** and zeta potential **(c)** for assessing the stability in solution for Se-NPs. [22].

The average particle size determined by Scherrer's equation was about 31 nm. The diffraction peak of Se-NPs obtained was well defined and was in agreement with the standard XRD patterns of Se-NPs (JCPDS-06-0362). Zeta potential analysis revealed the Se-Nps had a potential of -50 ± 07 mV indicating high stability in aqueous solution and aggregation effect in aqueous solution (Milli-Q water) [22].

## 4.2. Survivability & Wellbeing

The mean survivability of tilapia was 100%, 97.62%, 95.37%, and 54.73 % for the control group and dietary Se-NP supplemental doses of 0.5, 1.0 and 2.0 mg/kg, respectively (Table 1).

Table 1. Mean values ± Standard Deviations (SD) and ranges for biometric data of Nile tilapia fingerlings fed on different levels of Se-NP inclusions.

Dietary supplements	Length (cm)	Weight (gm)	Percentage Survivability

|  | Mean ± SD | Ranges |  | Mean ± SD | Ranges |  |  |
| --- | --- | --- | --- | --- | --- | --- | --- |
| Control | 6.81 ± 0.69 | 5.70 -7.90 |  | 7.57 ± 1.68 | 4.60 - 10.10 | 100.00 |  |
| 0.5 mg/Kg | 7.03 ± 0.13 | 5.50 - 8.00 |  | 8.36 ± 1.66 | 4.08 - 11.08 | 97.62 |  |
| 1 mg/Kg | 8.42 ± 0.40 | 7.30 - 9.10 |  | 16.09 ± 2.28 | 12.00 - 19.79 | 95.37 |  |
| 2 mg/Kg | 5.82 ± 0.43 | 5.11 - 6.90 |  | 4.74 ± 1.04 | 3.08 - 7.00 | 54.73 |  |

The survival rate was the lowest in the 2 mg/kg inclusion, compared to other groups (p < 0.05). The well-being of the fish was determined as Fulton's condition factor for each treatment. The condition factor (FCF) for the *O. niloticus* is shown in Table 2. The condition factor, K computed were 2.40, 2.40, 2.68, and 2.39 respectively for fish fed on nil, 0.5, 1.0, and 2.0 mg/Kg Se-Nps. The highest K value was observed for the 1 mg/kg treatment and the lowest was that obtained for the 2 mg/Kg treatment (Table 2).

Table 2. Coefficient of determination (R^2^), Regression of coefficient (b), Condition Factor (k), Relative Growth Rate (RGR in %), Specific Growth Rate (SGR in %), and Feed Conversion Rate (FCR) for the experimented Nile tilapia fingerlings fed on different levels of Se-NP inclusions

| Dietary Supplements | R^2^ | b | RGR (%) | SGR (%) | FCR | K |
| --- | --- | --- | --- | --- | --- | --- |
| Control | 0.74 | 2.08 | 680.41 | 3.42 | 2.53 | 2.35 |
| 0.5 mg/Kg | 0.89 | 2.35 | 770.83 | 3.61 | 2.09 | 2.37 |
| 1 mg/Kg | 0.95 | 2.81 | 1576.04 | 4.70 | 1.07 | 2.78 |
| 2 mg/Kg | 0.68 | 2.46 | 383.67 | 2.63 | 2.43 | 2.26 |

## 4.3. Length-Weight Relationship Dynamics & Growth Performance

The mean lengths and weights were significantly high in the 1mg/kg inclusion group compared to other groups (p< 0.05, Table 1). On the contrary, significantly weak growth outcomes were obtained in the 2 mg/kg inclusion group. Fish fed 1mg/kg Se-Np were observed to comprise the tallest (9.10 cm) and the heaviest (19.79 gm) individual, whereas the smallest fish was 5.11 cm in length and 3.08 gm in weight and found in 2 mg Se-NP supplemented group (Table 1).

Statistical analysis revealed significant variations (p < 0.05) in the average weight and length among all the treatments (Table 2). The value of the regression coefficient obtained from the LWR was 0.74, 0.89, 0.91, and 0.68 for fish fed on different dietary Se-NP inclusion levels 0.0 (control), 0.5, 1.0, and 2.0 mg/kg, respectively. There was a significant correlation between length and weight (p < 0.05). All the treatment groups showed a steady increase in weight following exposure to their respective treatments. Significantly, increased mean weights were obtained in fish fed with 1 mg Se-NP, followed by 0.5 gm Se-NP in comparison to the other groups (p < 0.05). As presented in table 2, the results of the current study showed that all the treatments, except 1 mg/kg, and the control groups demonstrated negative allometric growth (b < 2.5). Fish exposed to 1 mg/kg Se-Nps inclusions gave a “b” value close to 3 (b > 2.5) representing isometric growth. The growth performance of *O. niloticus* differed significantly (P < 0.05) in terms of weight, RGR, and SGR (Table 2). Feed performances of the fish fed the experimental diets are presented in Table 2. Feed conversion ratios (FCR) were assessed based on the feed consumption per treatment (considering the live fish in each treatment) throughout the whole research period. Treatment at 1 mg N-Se/kg showed higher RGR (1576.04), and SGR (4.70). Relatively poor FCR was observed in the control group compared to the other treatments; the highest FCR (1.07) being recorded for the 1 mg N-Se/ kg inclusion (Table 2).

## 4.4. Proximate composition of fish body

The average protein content of the basal feed used for all the treatments was 50.72 %. Proximate composition analysis of the control and treated fish groups is presented in Table 3. Body moisture, crude protein, crude lipid, crude fiber, and ash did not differ among treatments. However, the protein content for all the treatments is high, and that for fish exposed to 1 mg NSe showed relatively higher protein content than the other treatments.

Table 3. Proximate composition of fish whole body at the end of the experiment for the different treatment types.

| **Treatments** | **Moisture** | **Crude Protein** | **Crude Fat** | **Ash** |
| --- | --- | --- | --- | --- |
| **Control** | 77.82 | 71.75 | 13.77 | 11.66 |
| **0.5 mg/kg** | 79.39 | 73.15 | 16.23 | 13.03 |
| **1 mg/kg** | 77.6 | 79.8 | 13.43 | 12.02 |
| **2 mg/kg** | 77.83 | 77.35 | 13.2 | 12.11 |

## 4.5. Level of Selenium

The level of selenium in the fish blood, muscle, and liver following the results were obtained using Flameless Atomic Absorption Spectrophotometry (FAAS). Significant differences were observed in the levels of Se in the different inclusions of blood and liver tissue among 2 mg/kg supplemented group compared to other groups (p < 0.05), (Table 4), which followed an increasing trend through increment with an increase in Se concentrations.

Table. 4 Level of Selenium (mg/kg) in the liver, muscle tissue, and blood of fish at the end of the experiment for the different types of Se-NP inclusions and the control group.

**Control	0.5 mg/Kg	1 mg/Kg	2 mg/Kg**

**LIVER**	0.61	0.7	0.77	0.86

**TISSUE**	0.07	0.11	0.11	0.11

**BLOOD**	0.011	0.016	0.016	0.04

## 4.6. Fatty Acid Analysis

### 4.6.1. Fatty acid profile analysis

GC-MS analysis revealed the distribution of 22 fatty acids found in the tilapia samples, of which seven were SAFAs, five were MUFAs, and five were PUFAs (Table 5). The results were expressed in terms of the relative peak area/percentage of abundance. PUFA content was higher than the MUFA and the SAFA for all the experimental and the control groups. Importantly, omega -3 fatty acid was significantly high (∑ n-3 = 9.05) in 1 mg/kg N-Se inclusion group compared to that of any other treatments including the control group (Fig 2), whereas omega-6 fatty acids were lower (∑ n-6 = 27.42) in 1 mg/kg Se-Nps inclusion group in comparison to other groups (Fig 2, Table 5 ).

Figure 2. Percent abundance of representative essential omega 3 (n-3; DHA, docosahexaenoic acid and EPA, eicosapentaenoic acid) and omega 6 (n-6; DGLA, eicosatetraenoic acid and AdA, eicosatrienoic acid) fatty acids among all the groups, control; 0.5 mg Se-Np; 1 mg Se-Np; 2 mg Se-Np groups, depicting 1 mg/kg SeNp group possessing significantly high n-3 fatty acids and low n-6 fatty acids (p < 0.05, ANOVA test), in comparison to other treatments.

The total PUFA content was significantly high (38.47, p < 0.05) in 1 mg/kg SeNP inclusion group, while SAFA content (26.03) was significantly low (p < 0.05) in 1 mg/kg SE-Np group compared to other groups (Table 5).

**Table 5**. Percentage of abundance of the FAs with reference to the contribution of the essential fatty acid in accordance with the saturation groups within and across the treatment groups.

| Formula | Saturation (C:D form) | Fatty Acid Groups | % Peak Area\Abundance | | | |
| --- | --- | --- | --- | --- | --- | --- |
|  |  |  | **Control** | **0.5 mg** | **1 mg** | **2 mg** |
| C13H26O2 | C12:0 | SAFA | 0.23 | 0.15 | 0.1 | 0.29 |
| C15H30O2 | C14:0 | SAFA | 1.99 | 1.8 | 1.7 | 1.98 |
| C16H32O2 | C 15: I | SAFA | 1.32 | 0.44 | 0.39 | 1.42 |
| C16H32O2 | C15:0 | SAFA | 0.69 | 0.74 | 0.4 | 0.42 |
| C17H34O2 | C 16:0 | SAFA | 19.97 | 18.37 | 18.21 | 19.99 |
| C17H32O2 | C 16: 1(n7) | MUFA | 6.34 | 4.85 | 4.73 | 6.6 |
| C18H36O2 | C17:0 | SAFA | 0.81 | 0.9 | 0.78 | 0.87 |
| C19H38O2 | C18:0 | SAFA | 6.74 | 6.61 | 5.45 | 6.99 |
| C19H36O2 | C 18:1 *cis(n9)* | MUFA | 20.31 | 22.46 | 23.87 | 23.38 |
| C19H36O2 | C 18:1 *trans(n9)* | MUFA | 3.75 | 3.45 | 3.19 | 3.22 |
| C19H34O2 | C 18:2 *(n6)* | PUFA | 27.57 | 25.17 | 24.71 | 26.49 |
| C19H32O2 | C 18: 3 (n3) | PUFA | 3.44 | 3.55 | 3.65 | 2.33 |
| C21H40O2 | C 20:1 (n9) | MUFA | 0.55 | 0.68 | 0.86 | 0.84 |
| C21H36O2 | C 20 :3 (n6) | PUFA | 1.13 | 1.11 | 1.09 | 1.27 |
| C21H34O2 | C 20 :4 (n6) | PUFA | 1.71 | 1.63 | 1.06 | 1.99 |
| C22H36O2 | C20 : 4 (n3) | PUFA | 0.25 | 0 | 0.33 | 0.33 |
| C21H32O2 | C 20: 5 (n3) | PUFA | 0.28 | 0.29 | 0.29 | 0.21 |
| C23H38O2 | C 22:4 (n6) | PUFA | 0.52 | 0.52 | 0.26 | 0.59 |
| C23H36O2 | C 22: 5 (n6) | PUFA | 0.65 | 0.64 | 0.3 | 0.88 |
| C23H36O2 | C 22: 5 (n3) | PUFA | 0.2 | 1.16 | 1.21 | 1.01 |
| C23H34O2 | C 22: 6 (n3) | PUFA | 2.98 | 3.35 | 3.57 | 0.97 |
| Unidentified | | | 1.03 | 1.16 | 0.94 | 1.02 |
| ∑ SAFA | | | 31.75 | 29.01 | 26.03 | 31.96 |
| ∑ MUFA | | | 31.95 | 31.44 | 32.65 | 34.04 |
| ∑ PUFA | | | 34.73 | 35.42 | 38.47 | 32.07 |
| ∑ n-6 | | | 31.58 | 29.07 | 26.42 | 31.22 |
| ∑ n-3 | | | 7.15 | 7.35 | 9.85 | 4.85 |

These findings were further confirmed by the mass spectrometrogram, including DHA, 22:6 (n-3) and (EPA), 20:5 (n-3) as the representative omega-3 fatty acids, and AdA 22:4 (n-6) and DGLA 20:3 (n-6) as the representative omega-6 fatty acids, which depicted the abundance of the n-3 and n-6 fatty acids was considerably high and low in the 1 mg Se-Np group, respectively (Fig. 3-6).

Figure 3. Mass Spectrometry Plot: Counts vs. mass-to-charge (m/z) assessed using GC/MS Mass hunter: Representative plot for omega-3 depicts DHA, C 22:6 (n-3), for the different treatments: (A), Control; (B), 0.5 mg/kg Se-Np; (C), 1 mg/kg Se-Np; (D), 2 mg/kg Se-Np inclusions. The different treatment samples for the given fatty acid depicted most of the characteristic indexed peaks for the fatty acids, with highest peaks as the main representative of the fatty acid with similar molecular ion peak (m/z).

Figure 4. Mass Spectrometry Plot: Counts vs. mass-to-charge (m/z) assessed using GC/MS Mass hunter: Representative plot for omega-3 depicts EPA, C 20:5 (n-3) for the different treatments: (A), Control; (B), 0.5 mg/kg Se-Np; (C), 1 mg/kg Se-Np; (D), 2 mg/kg Se-Np inclusions. The different treatment samples for the given fatty acid depicted most of the characteristic indexed peaks for the fatty acids, with the highest peaks as the main representative of the fatty acid with a similar molecular ion peak (m/z).

Figure 5. Mass Spectrometry Plot: Counts vs. mass-to-charge (m/z) assessed using GC/MS Mass hunter: Representative plot for omega-3 I depicts AdA, 20:4 (n-6) for the different treatments: (A), Control; (B), 0.5 mg/kg Se-Np; (C), 1 mg/kg Se-Np; (D), 2 mg/kg Se-Np inclusions. The different treatment samples for the given fatty acid depicted most of the characteristic indexed peaks for the fatty acids, with highest peaks as the main representative of the fatty acid with similar molecular ion peak (m/z).

Figure 6. Mass Spectrometry Plot: Counts vs. mass-to-charge (m/z) assessed using GC/MS Mass hunter: Representative plot for omega-3 depicts DGLA, 20:3 (n-6) for the different treatments: (A), Control; (B), 0.5 mg/kg Se-Np; (C), 1 mg/kg Se-Np; (D), 2 mg/kg Se-Np inclusions. The different treatment samples for the given fatty acid depicted most of the characteristic indexed peaks for the fatty acids, with the highest peaks as the main representative of the fatty acid with a similar molecular ion peak (m/z).

### 4.6.2. Nutritional Quality Indexes (NQI)

Different fats and fatty acids have varying quality indices for assessing their dietary benefits. In this study, a combination of indexes was utilized to conduct a comprehensive evaluation of the nutritional values of the fish tissue after being exposed to different Se-Nps inclusion levels (Fig 7). Ratios of PUFA/SAFA and n-3/n-6 were found to be 1.35 and 0.33, respectively in the fish fed 1 mg /kg Se-Nps inclusion, which was relatively higher compared to other treatments, including the control group. In addition, fish fed 1 mg/kg Se-Np exhibited lower ratios of n-6/n-3 (3.03). The atherogenicity index IA, and thrombogenicity index IT were relatively low at 0.36 and 0.44, respectively in the 1 mg/kg Se-Nps inclusion group compared to other treatments including the control (Fig. 7).

Figure 7. Nutritional Quality Indexes (NQI): n-6/n-3, PUFA/SAFA. IT, and IA for Control (A), 0.5 mg Se-Np/kg (B), 1 mg Se-Np/kg (C), and 2 mg Se-Np/kg (D) inclusions.

# 5. Discussion

Aquaculture improvement is currently been pursued in different dimensions for addressing the needs of malnutrition and providing adequate nutrition to the entire community, which involves the inclusion of Nano-nutrients, fermented materials, trace elements, and many others [25]. The use of nanoparticles of trace elements is one of the promising approaches for feed supplementation in a variety of fish, which serves as a handy way of delivery and easy availability to the fish. The present study showed that the administration of a specific dose of SeNPs enhanced the growth performance and nutrient quality index in Nile tilapia that serves as one of the staple fish food across the continents and can meet the nutrition requirement of the growing population. In the present study, the experimental and the control groups showed negative allometric growth (b < 3); however, tilapia fish fed 1 mg/kg of Se-NP inclusion showed nearly isometric growth with b value of 2.81. Previous studies have reported significantly higher growth in terms of weight gain in the diet containing 1 mg/kg of Se-Np in fish, which was in line with our findings [25].

The present study revealed that 1 mg/kg Se-Nps supplementation resulted in significantly high weight gain (> 210 %, p >0.05) compared to other groups, which was in line with other studies performed in crucian carp [26] and rainbow trout [27]. Selenium is a vital and key element of fish nutrition and is essential for the functioning of an array of Se-carrying proteins, called sialoproteins [28, 29]. Nanoselenium has improved bioavailability because of its extraordinarily small size, such that it can be easily absorbed and be readily uptaken by the cells, thereby enhancing the metabolic and physiological activities [30].

Nile tilapia demonstrated enhanced growth performance upon nanoselenium supplementation. Growth parameters RGR and SGR values were significantly high and low in the 1 mg/kg and 2 mg/kg Se-Np inclusion groups, respectively in comparison to others. The low growth performance in 2 mg/kg Se-Np inclusion group could be attributed to the toxic effect of the over-dose supplementation of Se-Nps. In addition, the fish fed 1 mg/kg Se-Nps revealed better k values (condition factor) than the other treatment groups. Several studies also reported low FCR, SGR, and RGR obtained for 2 mg/kg Se-Nps in different fish varieties [5, 31]. Fish fed 1 mg/kg N-Se had by far lower FCR (1.07) than the control group (2.53) (*p* < 0.05*).* According to [32], the higher the FCR, the lower is the growth efficiency and inverse is the link between SGR and FCR. The result from our study is in agreement with these findings.

However, some studies reported enhanced growth performance at higher Se-Nps supplementation dose, such as at 3.67 mg/kg Se inclusion in African catfish [33]. On the contrary, the optimum Se-Np dose concentration was low for rainbow trout (0.38 mg/kg) [34], channel catfish (0.25 mg/kg) [35], and for groupers (0.77 mg/kg) [36]. Such findings demonstrate that the optimum dose of Se-Np supplementation for enhancing growth and productivity varies with fish type [37], which could be attributed to the bioaccumulation or bioavailability of Se inside the fish tissues.

Selenium distribution varied across different tissues in the fish treatments. Se accumulation in the fish tissues was generally low and increased in a concentration-dependent manner. Se levels in the body tissue was 0.055 mg/kg in our study, which was lower than that obtained for catfish (0.87 mg/kg) [37]. According to the recommended dietary allowance (RDA) of selenium for humans, the maximum concentration is 55 µgm selenium per day [38]; which agreed with our result (0.055 mg/kg ~ 55 µmg/kg). In addition, a slightly higher and dose-dependent increment of Se levels was observed in the liver with an increase in inclusion concentration. Se level in the blood samples were similar for the 0.5 and the 1 mg/kg inclusions, while Se level increased significantly in the 2 mg/kg treatment group. The observed difference in the accumulation of Se within the particular organ/tissue could be attributed to the difference in their physiological functions and increased dose of Se-Np exposure. The liver is the most important target organ involved in the metabolism and detoxification of xenobiotics in vertebrates [39]. Because fish were not starved prior to sampling, livers were observed to contain high fat [40]. The low Se level in the blood samples could be because blood is involved in transporting, and not accumulating Se, from the digestive system to other organs, specifically to the liver, wherein accumulation and bio-magnification of selenium [41, 34] occurs.

Another intriguing feature of growth performance was the content of different fatty acids (FA) in the fish. Among the different FAs analyzed, the SAFA C 16:0 and C 18:0, MUFA C 18: 2 (n6) , C 18:1 (cis n9), C 18: 1 (trans n9) and C: 16: 1 (n7), PUFA C 18:2 (n6), C 18: 3 (n3), and C 22: 6 (n3) showed varying percentages of abundance across the treatment groups (Fig. 4, and Supplementary Table-1). Previous studies suggest that diets rich in saturated fatty acids are not healthy, and the consumption of mono and polyunsaturated fatty acids is recommended to maintain good health [42, 43]. Generally, the good thing is fish species have a relatively low SAFA [44], which have been linked to coronary heart disease [45]. Many studies have shown that fish oil has a high nutritional value because it is rich in PUFAs, which consist of n-3 PUFAs and n-6 PUFAs, particularly docosahexaenoic acid (DHA) and eicosapentaenoic (EPA) [46-48]. In this study, the fatty acids were observed in order: PUFA > MUFA> SAFA, in mainly the 1 mg/kg Se-Np inclusion group, which could be benefitting for the health of humans. PUFA/SAFA and n3/n6 ratios are considered to be important parameters correlated to the nutritional value of food types for human health. According to the WHO report, the minimum value for PUFA: SAFA is 0.45 or more [49]. Our findings revealed overall PUFA:SAFA greater than 0.45, which was further significantly high in the fish fed 1 mg/kg Se-Np inclusion compared to other groups.

The benefits of healthy fats, ω-3 (n-3) fatty acids, such as ALA, EPA, and DHA to humans include prevention of cardiovascular disease, improvement in mental health, and in vision-related issues [50,51]. DHA can significantly promote an infant’s intellectual development, enhance memory, and improve eyesight [52]. EPA can prevent atherosclerosis, hypertension, and allergic reactions [53]. In the present study, fish fed with 1 mg/kg Se-Np inclusion showed higher levels of DHA and EPA, in addition to significantly high omega-3 content (n-3 = 9.37) in comparison to other treatments and control, which could be thus attributed to the effect of Se-Np supplementation at the optimum concentration (1 mg/kg). Such finding was in agreement with previous studies, which reported that Se-Np was effective in improving the productive performance and glutathione peroxide (GSH-Px) activity of enzymes responsible for improving the fatty acid profile and antioxidant potential [54].

Furthermore, n-3/n-6 ratios, which depict the nutritional quality indices, were relatively low in the treatment groups of this study, which were in line with previous studies [55], and have been known to possess health-promoting effects. Conversely, diets high in n-6 fats (high dietary n-6/n-3 ratio) lead to human health deficits including inflammation, asthma, and reduced kidney function [55, 56], which were low in the 1 mg/kg Se-Np inclusion group. According to the UK Department of Health, a ratio value for the n-6 to n-3 below 4.0 is recommended for cardiovascular disease prevention [57], which was recorded to be 3.0 in the 1 mg/kg treatment group and significantly low compared to other treatment groups. Similarly, the other important NQI: IA and IT were considerably low in 1 mg/kg Se-Np inclusion group, which corroborated with previous studies [58, 59]. Generally, fish fed with 1 mg/kg Se-Np inclusion demonstrated the highest weight and length gain, exhibited high nutritional quality meat, in addition to higher levels of n-3 fatty acids, all of which could be because of the optimum dose of Se-Np supplementation leading to higher bioavailability and metabolism in tilapia (*O. niloticus*).

Overall, the study reveals the growth-promoting effects and the improved nutritional quality of Nile tilapia upon nanoselenium supplementation. Nanonutrient supplementation is now considered one of the promising approaches for enhancing aquaculture, and this study has elaborated such effects using Se-Nps in Nile tilapia. Nano-nutrient supplementation at an appropriate dose could be a promising approach for enhancing aquaculture in a variety of commercial fish and could address the issues of malnutrition of adequate nutrition across the globe.

# 6. Conclusion

Nanoselenium supplementation enhanced the growth performance and nutrition quality of Nile tilapia at a specific dose of 1 mg/kg. As selenium has several key roles in the productivity and immune status of an organism, nanosupplementation of selenium increased the bioavailability and overall growth parameters like length, weight, omega 3 fatty acid content, and overall health of tilapia. The findings stress the importance of the production of value-added fish meat with the inclusion of vital micronutrient, selenium that could aid in ameliorating selenium deficiency in the population. Industries related to fish farming could explore the development of application of feed supplementations with nanoselenium at optimum levels as substantial input for enhancing the growth performance and productivity of tilapia such that they can meet the growing demand of fish as a staple diet among the modern population.

# Acknowledgments

The authors extend their appreciation to the deanship of scientific research for funding this article by Taif University Researchers Supporting Project number (TURSP-2020/203), Taif University, Taif, Saudi Arabia. In addition, the support of the Ethiopian Biotechnology Institute (EBTI) is gratefully acknowledged for assistance for the research work.

# Reference

1. FAO. The State of World Fisheries and Aquaculture 2020. Sustainability in action; FAO: Rome, Italy, 2020. Rome.<https://doi.org/10.4060/ca9229en>.
2. Gibtan A, Getahun A, Mengistu S. Effect of stocking density on the growth

performance and yield of Nile tilapia [*Oreochromis niloticus* (L., 1758)] in a cage

culture system in Lake Kuriftu, Ethiopia. J. Aquaculture Research*.* 2008**;** 39: 1450-

1460. doi:10.1111/j.1365-2109.2008.02021.x.

3. FAO. FAO Global Fishery and Aquaculture Production Statistics 1950-2017. Food and Agriculture Organization of the United Nations. Fishery Statistical Collections. Global Production e-bulletin. 2019a. <http://www.fao.org/fishery/statistics/global-aquaculture-production/query/en>.
4. FAO. Top 10 species groups in global aquaculture 2017. WAPI factsheet. Rome. 2019b. <http://www.fao.org/3/ca5224en/CA5224EN.pdf>.
5. Iqbal S, Atique U, Mughal MS, Younus M, Rafique MK, Haider MS, et al. Effect of supplemental selenium in fish feed boosts growth and gut enzyme activity in juvenile tilapia (*Oreochromis niloticus*). Journal of King Saud University*–* Science. 2020; 32: 2610-2616.
6. Radhi AM, Fazlinda M-FN, Amal MNA, Rohasliney H. A review of length-weight relationships of freshwater fishes in Malaysia. Transylvanian Review of Systematical and Ecological Research. 2018; 20: 55-68. DOI: 10.1515/trser-2018-0005.
7. Blackwell BG, Brown ML, Willis DW. Relative Weight (Wr): Status and current use in fisheries assessment and management. Reviews in Fisheries Science. 2010; 8: 1–44. <https://doi.org/10.1080/10641260091129161>.
8. El-Sayed MM, Ezzat AA, Kandeel KM, Shaban FA. Biochemical studies on the lipid content of Tilapia nilotica and Sparus auratus. Comp. Biochem. Physiol.

1984; 79 B: 589–594. DOI: [10.1016/0305-0491(84)90371-7](https://doi.org/10.1016/0305-0491(84)90371-7).

9. Ahlgren G, Blomqvist P, Boberg M, Gustafsson I.-B. Fatty acid content of the dorsal muscle – an indicator of fat quality in freshwater fish. J. Fish Biol*.* 1994**;** 45: 131–157. <https://doi.org/10.1111/j.1095-8649.1994.tb01292.x>.
10. Zenebe T, Ahlgren G, Gustafsson I.-B, Boberg M. Fatty acid and lipid content of *Oreochromis niloticus* L. in Ethiopian lakes – dietary effects of phytoplankton. Ecol. Freshw. Fish. 1998a; 7: 146–158.
11. Zenebe T, Ahlgren G, Boberg M. Fatty acid content of some freshwater fish of commercial importance from topical lakes in the Ethiopian Rift Valley. J. Fish. Biol 1998b**;** 53: 987–1005.
12. Sokoła-Wysoczańska E, Wysoczański T, Wagner J, Czyz K, Bodkowski R, Lochynski S, et al. Polyunsaturated Fatty Acids and Their Potential Therapeutic Role in Cardiovascular System Disorders-A Review. Nutrients. 2018; 10(10):1561. doi:10.3390/nu10101561
13. Abedi E, Sahari MA. Long-chain polyunsaturated fatty acid sources and evaluation of their nutritional and functional properties. Food Sci Nutr. 2014; 2(5):443-463. doi:10.1002/fsn3.121.
14. Khosravi-Katuli K, Prato E, Lofrano G, Guida M, Vale G, Libralato G. Effects of nanoparticles in species of aquaculture interest. REVIEW ARTICLE. Environ Sci Pollut Res Int. 2017; 24(21):17326-17346. doi: 10.1007/s11356-017-9360-3.
15. Vinceti M, Filippini T, Del Giovane C, Dennert G, Zwahlen M, Brinkman M, et al. Selenium for Preventing Cancer. Database Syst. Rev*.* 2018; 29: 6491296. 10.1002/14651858.CD005195.pub4.
16. Ellis D, Sors TG, Brunk DG, Albrecht C, Orser C, Lahner B, et al. Production of Se-Methyl Selenocysteine in Transgenic Plants Expressing Selenocysteine Methyl Transferase. BMC Plant Biol*.* 2004; 4: 1. 10.1186/1471-2229-4-1.
17. Xuxia Zhou, Yanbo Wang, Qing Gu, Weifen Li. Effects of different dietary selenium sources (selenium nanoparticle and selenomethionine) on growth performance, muscle composition and glutathione peroxidase enzyme activity of crucian carp (*Carassius auratus Gibelio*), Aquaculture. 2009; 291: 78–81. 10.1016/j.aquaculture.2009.03.007.
18. Liu LW, Liang X.-F, Li J, Fang JG, Yuan XC, Li J, et al. Effects of Dietary Selenium on Growth Performance and Oxidative Stress in Juvenile Grass carp, *Ctenopharyngodon Idellus*. Aquacult Nutr. 2018; 24: 1296–1303. doi:10.1111/anu.12667.
19. Sarkar B, Bhattacharjee S, Daware A, Tribedi P, Krishnani KK, Minhas PS. Selenium Nanoparticles for Stress-Resilient Fish and Livestock. Nanoscale Res Lett. 2015; 10(1):371. doi:10.1186/s11671-015-1073-2.
20. Ashouri S, Keyvanshokooh S, Salati AP, Johari SA, Pasha-Zanoosi H. Effects of Different Levels of Dietary Selenium Nanoparticles on Growth Performance, Muscle Composition, Blood Biochemical Profiles and Antioxidant Status of Common Carp (*Cyprinus carpio*). Aquaculture. 2015; 446: 25–29. doi:10.1016/j. aquaculture. 2015.04.021.
21. Ilham I, Fotedar R. Growth, Enzymatic Glutathione Peroxidase Activity and Biochemical Status of Juvenile Barramundi (*Lates calcarifer*) Fed Dietary Fermented Soybean Meal and Organic Selenium. Fish. Physiol. Biochem. 2017; 43: 775–790. doi:10.1007/s10695-016-0331-2.
22. Fasil DM, Hamdi H, Al-Barty A, Zaid AA, Parashar SKS, Das B. Selenium and Zinc Oxide Multinutrient Supplementation Enhanced Growth Performance in Zebrafish By Modulating Oxidative Stress And Growth-Related Gene Expression. Front.Bioeng.Biotechnol*.* 2021; 9: 721717. doi: 10.3389/fbioe.2021.721717.
23. FAO. 2015. A Guide to Recirculation Aquaculture- RAS. Food and Agriculture Organization- FAO. 2015. Rome, Italy. <http://www.fao.org>.
24. Ulbricht TLV, Southgate DAT. Coronary heart disease: seven dietary factors. Review Article. THE LANCET. 1991**;** 338: 8773-985-992.

DOI:<https://doi.org/10.1016/0140-6736(91)91846-M>

25. Rathore SS, Murthy HS, Girisha SK, Nithin MS, Nasren S, Mamun MAA, et al. Supplementation of nano‐selenium in fish diet: Impact on selenium assimilation and immune-regulated selenoproteome expression in monosex Nile tilapia (*Oreochromis niloticus*).Comparative Biochemistry and Physiology Part C: Toxicology & Pharmacology. 2021**;** 240: 108907. DOI: [10.1016/j.cbpc.2020.108907](https://doi.org/10.1016/j.cbpc.2020.108907).
26. Zhou X, Wang Y, Gu Q, Li W. Effect of different dietary selenium source (selenium nanoparticle and selenomethionine) on growth performance, muscle composition and glutathione peroxidase enzyme activity of crucian carp (*Carassius auratus gibelio*). Aquaculture. 2009**;** 291: 78-81.
27. Hunt AO, Berkoz M, Ozkan F, Yalin S, Ercen Z, Erdogan E, Gunduz SG. Effects of Organic Selenium on Growth, Muscle Composition, and Antioxidant System in Rainbow Trout. The Israeli journal of aquaculture- Bamidgeh, 2011; 63: 562, 10.
28. Hatfield DL, Gladyshe, VN. How selenium has altered our understanding of the genetic code. *Mol Cell Biol*. 2002**;** 22: 3565-76. doi: 10.1128/mcb.22.11.3565- 3576.2002.
29. Papp LV, Lu J, Holmgren A, Khanna KK. From selenium to selenoproteins: synthesis, identity, and their role in human health. Comprehensive Invited Review. Antioxid Redox Signal**.** 2007**;** 9: 775-806. doi: 10.1089/ars.2007.1528.
30. Fasil DM, Paritosh P, Parashar SKS, Biswadeep D. Mechanistic insights into diverse nano-based strategies for aquaculture enhancement: A holistic review. Aquaculture. 2020**;** 519: 734770. <https://doi.org/10.1016/j.aquaculture.2019.734770>.
31. El-Hammady AKI, Ibrahim SA, El-Kasheif MA. Synergistic reactions between vitamin E and Selenium in diets of hybrid tilapia (*Oreochromis niloticus ×	Oreochromis aureus*) and their effect on the growth and liver histological structure.

Egyptian Journal of Aquatic Biology and Fish. 2007**;** 30: 53-58.	DOI: [10.21608/ejabf.2007.1914](http://dx.doi.org/10.21608/ejabf.2007.1914).

32. Sonia Iqbal, UA, Shahid M, Muhammad SH, Hafiza SIl, Khalid AAl-Ghanim, et al. Effect of supplemental selenium in fish feed boosts growth and gut enzyme activity in juvenile tilapia (*Oreochromis niloticus*), Journal of King Saud University-Science. 2020; 32: 2610-2616. <https://doi.org/10.1016/j.jksus.2020.05.001>.
33. Abdel-Tawwab M, Mousa MAA, Abbass FE, Abbass E. Growth performance and physiological response of African catfish, *Clarias gariepinus* (B.) fed organic

selenium prior to the exposure to environmental copper toxicity. Aquaculture. 2007**;** 272 (1-4): 335–345DOI: [10.1016/j.aquaculture.2007.09.004](http://dx.doi.org/10.1016/j.aquaculture.2007.09.004).

34. Hilton JW, Hodson PV, Slinger SJ. Absorption, distribution, half-life and Possible routes of elimination of dietary selenium in juvenile rainbow trout (*Salmo gairdneri*). Comparative Biochemistry and Physiology Part C: Comparative Pharmacology**.** 1982**;** 71: 49-55. <https://doi.org/10.1016/0306-4492(82)90009-0>.
35. Gatlin DM, Wilson RP. Dietary Selenium Requirement of Fingerling Channel Catfish. The Journal of Nutrition*.* 1984**;** 114: 627–633. DOI: [10.1093/jn/114.3.627](https://doi.org/10.1093/jn/114.3.627).
36. Lin YH, Shiau SY. Dietary selenium requirements of juvenile grouper, *Epinephelus malabaricus.* Aquaculture. 2005**;** 250: 356-363.

<https://doi.org/10.1016/j.aquaculture.2005.03.022>.

37. Schram E, Pedrero Z, Cámara C, Van Der Heul JW, Joop B Luten JB, Enrichment of African catfish with functional selenium originating from garlic*.* 2008; 39(8), 850–860*.* doi:10.1111/j.1365-2109.2008.01938.x.
38. Institute of Medicine (US) Panel on Dietary Antioxidants and Related Compounds. Dietary Reference Intakes for Vitamin C, Vitamin E, Selenium, and Carotenoids. Washington (DC): National Academies Press (US); 2000. 7, Selenium. Available from: https://www.ncbi.nlm.nih.gov/books/NBK225470/
39. Curtis J. Omiecinski, John P. Vanden Heuvel, Gary H. Perdew, Jeffrey M. Peters, Xenobiotic Metabolism, Disposition, and Regulation by Receptors: From Biochemical Phenomenon to Predictors of Major Toxicities. Toxicological Sciences. 2011; 120: S49–S75,  <https://doi.org/10.1093/toxsci/kfq338>.
40. Stoneham TR, Kuhn DD, Taylor DP, Neilson AP, Smith SA, Gatlin DM, et al. Production of omega-3 enriched tilapia through the dietary use of algae meal or fish oil: Improved nutrient value of fillet and offal. PLoS ONE. 2018; 13(4): e0194241. https://doi.org/10.1371/journal. pone.0194241
41. Jaishankar M, Tseten T, Anbalagan N, Mathew BB, Beeregowda KN. Toxicity, mechanism and health effects of some heavy metals. Interdiscip Toxicol. 2014; 7(2):60-72. doi:10.2478/intox-2014-0009.
42. Hu FB, Stampfer MJ, Manson JE, Ascherio A, Colditz GA, Speizer FE, et al. Dietary saturated fats and their food sources in relation to the risk of coronary heart disease in women. American Journal of Clinical Nutrition**.** 1999**;** 70: 1001-1008.
43. Mensink RP, Zock PL, Kester AD, Katan MB. Effects of dietary fatty Acids and carbohydrates on the ratio of serum total to HDL cholesterol and on serum Lipids and apolipoproteins: a meta-analysis of 60 controlled trials. American Journal	of Clinical Nutrition. 2003**;** 77: 1146-1155.
44. Simopoulos AP. The importance of the ratio of omega-6/omega-3 essential fatty acids. Biomed Pharmacother. 2002**;** 56: 365-379. DOI: [10.1016/s0753-3322(02)00253-6](https://doi.org/10.1016/s0753-3322(02)00253-6)
45. Santos S, Oliveira A, Lopes C. Systematic review of saturated fatty acids on

inflammation and circulating	levels of adipokines. Nutr Res*.* 2013**;** 33: 687-695.

<https://doi.org/10.1016/j.nutres.2013.07.002>.

46. Tocher DR, Betancor MB, Sprague M, Olsen RE, Napier JA. Omega-3 Long-Chain Polyunsaturated Fatty Acids, EPA and DHA: Bridging the Gap between Supply and Demand. Nutrients. 2019; 11(1):89. doi:10.3390/nu11010089
47. Patterson E, Wall R, Fitzgerald GF, Ross RP, Stanton C. Health implications of high dietary omega-6 polyunsaturated Fatty acids. J Nutr Metab. 2012; 2012: 539426. doi:10.1155/2012/539426.
48. Das, UN. Estrogen, Statins, and Polyunsaturated Fatty Acids: Similarities in Their Actions and Benefits- Is There a Common Link? *Nutrition***.** 2002**;** 18: 178–188.

doi: 10.1016/s0899-9007(01)00719-5.

49. WHO. Community prevention and control of cardiovascular diseases. Report of a WHO Expert Committee. World Health Organ Tech Rep Ser*.* 1986; 732:1-62*.* PMID:

3085359.

50. Swanson D, Block R, Mousa SA. Omega-3 fatty acids EPA and DHA: health benefits throughout life. *Adv Nutr*. 2012; 3(1):1-7. doi:10.3945/an.111.000893.
51. Leaf A, Weber PC. Cardiovascular effects of n-3 fatty acids. New England. Journal of Medicine*.* 1988**;** 318: 549-571. DOI: [10.1056/NEJM198803033180905](https://doi.org/10.1056/nejm198803033180905).
52. Lauritzen L, Brambilla P, Mazzocchi A, Harsløf LB, Ciappolino V, Agostoni C. DHA Effects in Brain Development and Function. Nutrients. 2016; 8(1):6. doi:10.3390/nu8010006.
53. JR Nelson, O Wani, HT May, M Budoff. Potential benefits of eicosapentaenoic acid on atherosclerotic plaques. Vascular Pharmacology. 2017; 91: 1-9.
54. Nadia L, Radwan TA, Salah Eldin, El-Zaiat AA, Mostafa ASA. Effect of Dietary Nano-Selenium Supplementation on Selenium Content and Oxidative Stability in Table Eggs and Productive Performance of Laying Hens. International Journal of Poultry Science*.* 2015**;** 14: 161-176. DOI:10.3923/ijps.2015.161.176.
55. Zhang X, Ning X, He X, Sun X, Yu X, Cheng Y, et al. Fatty acid composition analyses of commercially important fish species from the Pearl River Estuary, China. PLoS ONE. 2020; 15(1): e0228276. <https://doi.org/10.1371/journal.pone.0228276>.
56. Valencak TG, Gamsjäger L, Ohrnberger S, Culbert NJ, Ruf T. Healthy n-6/n-3 fatty acid composition from five European game meat species remains after cooking. BMC Res Notes. 2015; 8:273. doi:10.1186/s13104-015-1254-1.
57. Cordain, L, Eaton SB, Sebastian A, Mann N, Lindeberg S, Watkins BA, et al. Origins and evolution of the Western diet: health implications for the 21st century. Am J Clin Nutr. 2005**;** 81: 341–354. DOI: [10.1093/ajcn.81.2.341](https://doi.org/10.1093/ajcn.81.2.341).
58. Molnar T, Biro J, Balogh K, Mezes M, Hancz C. Improving the Nutritional Value of Nile Tilapia Fillet by Dietary Selenium Supplementation. The Israeli Journal of Aquaculture-Bamidgeh, IJA. 2011**;** 64: 744,746.
59. Tonial I, Oliveira D, Coelho A, Matsushita M, Coró F, Souza N, et al. Quantification of Essential Fatty Acids and Assessment of the Nutritional Quality Indexes of Lipids in Tilapia Alevins and Juvenile Tilapia Fish (*Oreochromis niloticus*). Journal of Food Research. 2014**;** 3: 3.
